# Variation in Magnetic Memory Testing Signals and Their Relationship with Stress Concentration Factors During Fatigue Tests Based on Back-Propagation Neural Networks

**DOI:** 10.3390/ma18051008

**Published:** 2025-02-25

**Authors:** Huipeng Wang, Qiaogen Wang, Huizhong Liu

**Affiliations:** School of Mechanical and Electrical Engineering, Jiangxi University of Science and Technology, Ganzhou 341000, China; 15707963656@163.com (Q.W.), huizhong6@163.com (H.L.)

**Keywords:** magnetic memory testing signal, stress concentration factor, tension–tension fatigue test, characteristic extraction, back-propagation neural network

## Abstract

To investigate the relationship between metal magnetic memory testing (MMMT) signals and stress concentration factors (SCFs), four-level sinusoidal constant-amplitude load tension–tension fatigue tests were carried out on 45CrNiMoVA steel specimens with different SCFs. The normal component of MMMT signals, *H_p_*(*y*), was collected during the fatigue tests, and three characteristics were extracted and analyzed during the tests, including the peak-to-peak value of abnormal peaks (Δ*H_p_*(*y*)), the slope coefficient of the fitting line of *H_p_*(*y*) (*K*_1_), and the slope coefficient of the fitting line of *H_p_*(*y*) between abnormal peaks (*K*_2_), and a back-propagation (BP) neural network was developed to differentiate the SCF of the specimens. The results showed that both fatigue load and fatigue cycle number influenced MMMT signals, and the characteristics remained stable as the fatigue cycle number increased for the same fatigue load but increased significantly as fatigue load increased. In addition, all the characteristics increased as the distance between the scan line and the center line increased, but none of them could be used to differentiate the SCF of the specimens. With properly selected input vector and hidden nodes, the established BP neural network can quantitatively recognize the SCF of specimens.

## 1. Introduction

Research on structural fatigue failure has long attracted the attention of scientists and engineers to prevent the fatigue failure of mechanical components that would result in major economic losses or even the loss of life [1,2]. And one of the most effective methods for the fatigue life analysis of structural components is predicting the degree of stress concentration. Stress concentration was confirmed by a large number of fatigue damage and experimental results, and it refers to an increase in peak stress near abruptly changed cross-sections, such as holes, notches, cracks, and other changes, which is well above the average stress in a structure [3]. Stress concentration is one of the most important factors leading to fatigue failure as fatigue cracks normally initiate from stress concentration zones, which means that stress concentration zones are weak links in terms of fatigue life [4]. Therefore, it is very important and useful to effectively determine and monitor the stress concentration degree and the stress concentration zone for the fatigue life analysis of structural components, in particular the critical ones, as both high safety and economic requirements are required today, which also represents a major challenge for both scientific research and engineering applications [4,5].

Non-destructive testing (NDT) techniques, which can identify the degree of stress concentration and the defects of structural components over their entire lifetime, are always in demand. These traditional NDT techniques, i.e., ultrasonic testing, radiographic testing, eddy current testing, magnetic particle testing, and penetration testing, can only detect existing defects. However, these techniques cannot change anything in terms of the stress concentration, which is one of the main reasons for fatigue failure. In the late 1990s, metal magnetic memory testing (MMMT), a new NDT method proposed by Russian researchers, changed this situation [6,7]. The basic principle of MMMT is related to the magneto-mechanical effect, and it can facilitate the early diagnosis and pre-warning of dangerous work pieces and parts of ferromagnetic materials. Due to the good quality of portable devices, their ease of use and high sensitivity, and the fact that no special magnetizing devices are required [8], MMMT attracted a lot of attention. Recently, many researchers have investigated the variation in MMMT signals during the fatigue process. Chen et al. analyzed fatigue crack propagation using MMMT and found that MMMT could be used for the fatigue life prediction of structural alloy steel [9]. Zhou et al. developed an MMMT method to detect fatigue crack initiation and propagation behavior [10]. Tong et al. used MMMT for quantitative stress measurement under repetitive tensile load [11]. Su et al. predicted the fatigue life of locally corroded bridge steel by MMMT [12]. Zhao et al. used MMMT to inspect the four-point bending fatigue life of a Q345B steel beam [13]. Yang et al. investigated the cumulative ductile damage of a butt weld under low cycle fatigue by MMMT signals [14]. Arifin et al. characterized the effects of the stress ratio on fatigue crack propagation based on MMMT [15].

The above studies show that MMMT is an effective NDT method for the fatigue life prediction of ferromagnetics. However, it should be noted that these studies focus primarily on the crack propagation stage, and only very few studies investigated fatigue life before crack initiation. And it would be useful to describe the stress concentration degree quantitatively by MMMT, since fatigue cracks usually initiate at tress-concentrated zones. For this purpose, a fatigue test was carried out on specimens with different SCFs, and the normal component of MMMT signals, *H_p_*(*y*), was measured during the test. The results show that *H_p_*(*y*) has obvious non-linear characteristics with SCFs. Artificial neural networks (ANNs) were invented to solve various complex scientific and engineering problems. They offer the advantage of finding solutions for complex, non-linear, multi-dimensional functional relationships without making assumptions about their nature beforehand [16]. Therefore, a three-layer feed-forward back-propagation (BP) neural network was proposed and tested for the SCF differentiation of the specimens. The experimental results show the success of the proposed method’s utilization.

## 2. Experimental Methodology

### 2.1. Specimen Preparation

Samples made of 45CrNiMoVA steel was selected for the experiment in this study. This is a high-quality alloy steel with very high yield strength and ultimate strength, and it is commonly used in gears, main shafts, crankshafts, etc. Its chemical composition is as shown in Table 1, with a yield strength of 1323 MPa and a tensile strength of 1470 MPa.

**Figure 1:**
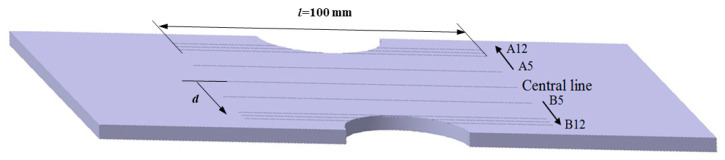
Sketch and measurement arrangement of tested specimens.

The specimens investigated were assumed to be tabular in structure with a length of 200 mm, a width of 30 mm, a thickness of 6 mm, and a surface roughness of 1.6 μm. Specimens were processed according to the Chinese national standard GB/T 3075-2021, as shown in Figure 1. Two pre-cut notches were machined symmetrically in the middle. Different notch sizes, radii R, widths 2a, and depths b mean different stress concentration factors (SCFs). The exact notch sizes with SCFs of 2, 3, 4, and 5 are shown in Table 2. For simplicity, the specimens with different SCFs are referred to as S2, S3, S4, and S5, respectively. In order to avoid the random error of the test, five specimens with different SCFs were used for the test.

To obtain a clean initial magnetic state, all specimens were heated at 850 °C for half an hour in a WCZ-30 vacuum heat treatment furnace with a vacuity of 8 × 10^−1^ Pa and then furnace-cooled to room temperature before the test.

Scan lines with a length of 100 mm were arranged parallel to the center line. The intervals between the scan lines and the center line were 5, 10, 11, and 12 mm in succession. For simplicity, the scan lines above the center line were labeled A5, A10, A11, and A12, and the ones below were labeled B5, B10, B11, and B12, which is also shown in Figure 1.

### 2.2. Experimental Instruments

A tension–tension fatigue test was carried out on a JNT150471 electro-hydraulic servo testing system (SANS Co., Ltd., Shenzhen, China) with the largest dynamic load of 50 kN, a dynamic error of 1%, and a load frequency from 0.01 to 40 Hz.

The normal component of MMMT signals, *H_p_*(*y*), was measured by an EMS-2003 metal magnetic memory apparatus (EDDYSUN (Xiamen) Electric Co., Ltd., Xiamen, China). The detection probe was fitted on a non-magnetic three-dimensional electrical control displacement instrument, which could be controlled to move automatically, to avoid interference from external magnetic fields and manual operating errors.

*H_p_*(*y*) signals were stored in the EMS-2003 metal magnetic memory apparatus at first, and then, the data could be transferred to the computer with the necessary software for EMS-2003. The characteristics of *H_p_*(*y*) signals were extracted using MATLAB (Matlab R2023a), and the following process, SCF recognition by the BP neural network, was also based on MATLAB.

### 2.3. Experimental Arrangement

Before the tension–tension fatigue test, each specimen was placed on the displacement instrument along the south–north axis, and the initial *H_p_*(*y*) of each scan line was measured by the EMS-2003 metal magnetic memory apparatus.

After that, each specimen was positioned vertically between the upper and lower grip holders of the testing machine, and tension–tension fatigue tests with four-level sinusoidal constant-amplitude loads with maximum loads of 10, 20, 30, and 40 kN, sequentially, were performed with a stress ratio of 0.1 and a frequency of 5 Hz. The specimen was taken off from the grip holders carefully after being loaded to a preset fatigue cycles, and the *H_p_*(*y*) signals of each scan line were measured in the same way as the initial signals. The specimen was then reloaded to another preset fatigue cycle number, and the above procedure was repeated. In the fatigue test, the fatigue cycle numbers of each load level were 1000, 3000, 6000, and 11,000, sequentially. When the fatigue cycle number was 11,000, the maximum load increased.

This experiment was carried out in a laboratory, and the specimens were far away from other ferromagnetic materials during the measurement. And the external magnetic field was a geomagnetic field only.

## 3. Results and Discussion

**Figure 2:**
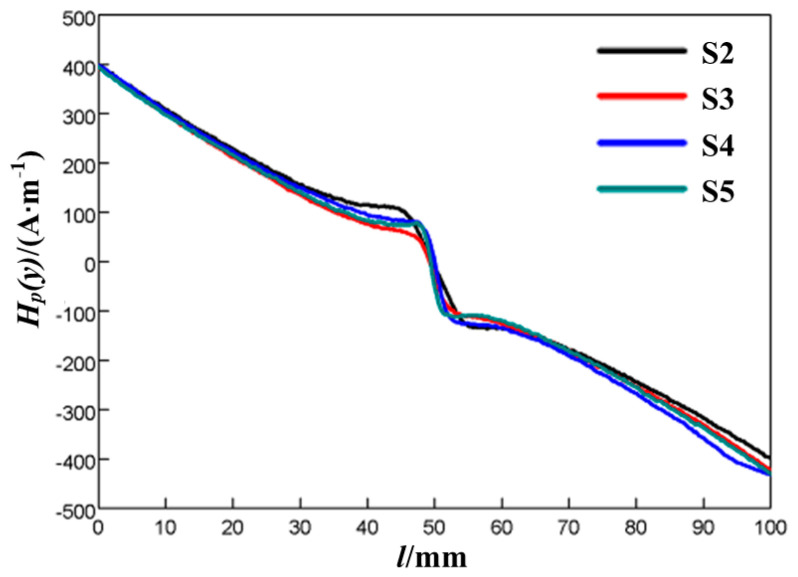
*H_p_*(*y*) of line A10 of different specimens with same fatigue load and fatigue cycle.

The results showed that the variation in the *H_p_*(*y*) of all scan lines and specimens corresponding to different fatigue loads and fatigue cycles presented regularity during the test: the left part of the signal was positive, and the left part was negative; the signal was linear beyond the pre-cut notch; and two abnormal peaks appeared around the notch, with a positive peak on the left and a negative peak on the right. Figure 2 shows the MMMT signals of line A10 of different specimens when the fatigue load was 10 kN and the fatigue cycle was 3000.

**Figure 3:**
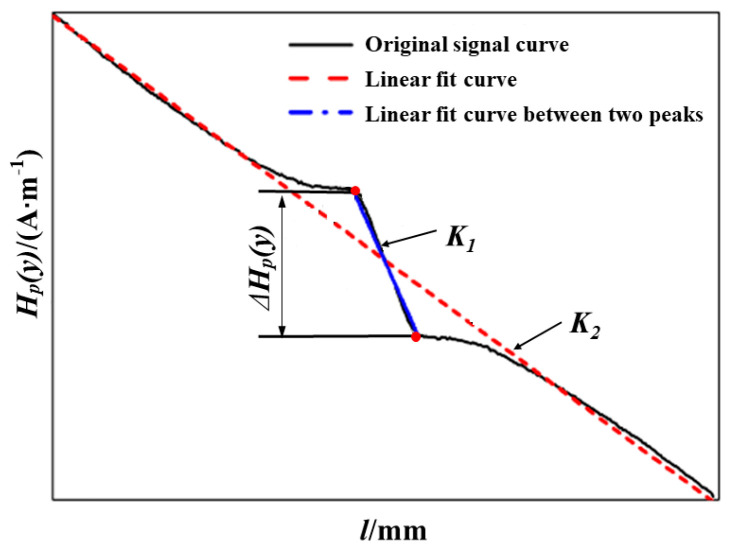
Sketch of characteristics, Δ*H_p_*(*y*), *K*_1_, and *K*_2_.

Three characteristics were presented and analyzed during the test, including the peak-to-peak value of abnormal peaks, the slope coefficient of the fitting line of *H_p_*(*y*), and the slope coefficient of the fitting line of *H_p_*(*y*) between abnormal peaks (referred to as Δ*H_p_*(*y*), *K*_1_, and *K*_2_ for short). Figure 3 shows a sketch of Δ*H_p_*(*y*), *K*_1_, and *K*_2_. It can be seen from Figure 3 that *K*_1_ and *K*_2_ are negative, and the absolute values were used for simplicity.

### 3.1. Effects of Fatigue Load and Fatigue Cycle on *Δ*H_p_(y), K_1_, and K_2_

**Figure 4:**
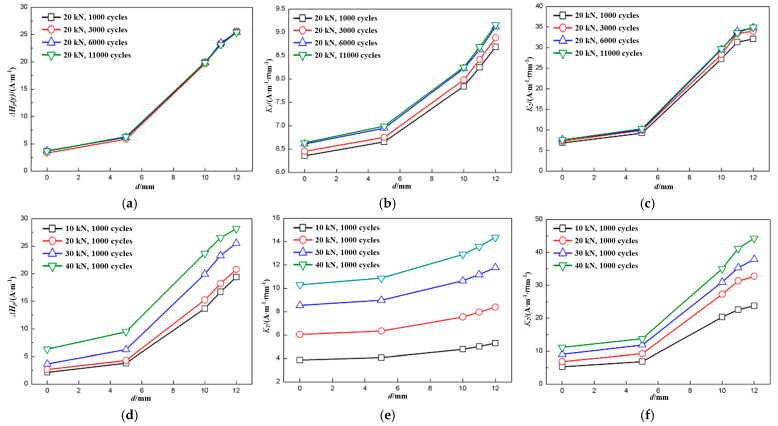
Characteristics of specimen *S2*: (**a**) Δ*H_p_*(*y*), (**b**) *K*_1_, and (**c**) *K*_2_ when fatigue load was 20 kN; (**d**) Δ*H_p_*(*y*), (**e**) *K*_1_, and (**f**) *K*_2_ when fatigue cycle number was 1000.

The Δ*H_p_*(*y*), *K*_1_, and *K*_2_ of each specimen had the same variation tendency, so only the characteristics of specimen S2 were presented and analyzed in this section. Figure 4 shows the characteristics of specimen S2 during different fatigue loads and fatigue cycles. The x-axis shows the distance between the scan line and the center line. To avoid experimental errors, the values were the average values of scan lines equidistant from the center line.

It can be seen from Figure 4 that Δ*H_p_*(*y*), *K*_1_, and *K*_2_ increased as the distance increased. When the fatigue load was 20 kN, they all changed only slightly with the increase in fatigue cycle number, as shown in Figure 4a–c, and it was difficult to tell them apart. The load has a much more pronounced effect on the characteristics when the fatigue cycle number was the same, as shown in Figure 4d–f, whose fatigue cycle number was limited to 1000. It was also found that all the characteristics, Δ*H_p_*(*y*), *K*_1_, and *K*_2_, increased as the fatigue load increased.

Stress applied to ferromagnetic material can change its magnetic behavior due to the movement of magnetic domains and the moment direction of the magnetic domains rotated in the direction of tensile stress and perpendicular to the direction of compression stress, which is also known as the piezo-magnetic effect [17]. The magnetization state of the ferromagnetics changed under axial tensile stress due to the piezomagnetic effect, and the change includes three phases: the reversible movement of the domain walls under a weak external magnetic field, the irreversible combination of the domain walls under an increasing magnetic field, and then the reversible rotation of the domain walls. According to reference [18], the irreversible part depends on the maximum applied stress. As soon as the axial tensile stress was applied, the specimen was magnetized by the stress-induced magnetic field along with the geomagnetic field, and the stress-induced magnetic field increased with an increase in the applied axial tensile stress, as did the irreversible part. Therefore, *H_p_*(*y*) signals increased dramatically as fatigue load increased. As the fatigue cycle increased, the irreversible part remained the same, and the magnetization state changed a bit due to fatigue damage accumulation. *H_p_*(*y*) signals increased a little when the fatigue cycle number increased at the same load, but they increased dramatically as the load increased. As the fatigue cycle number increased, all three characteristics, Δ*H_p_*(*y*), *K*_1_, and *K*_2_, therefore, changed only slightly, while they increased dramatically as fatigue load increased, as shown in Figure 4.

### 3.2. Effects of Stress Concentration Degree on *Δ*H_p_(y), K_1_, and K_2_

**Figure 5:**
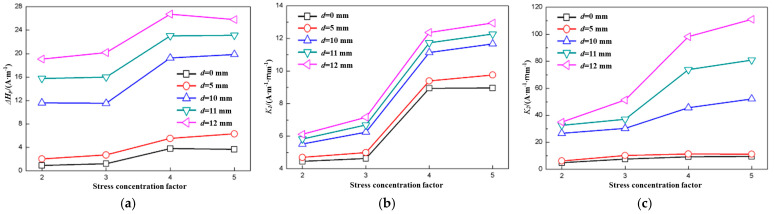
Characteristics of different specimens with fatigue load of 20 kN and fatigue cycle number of 11,000. (**a**) Δ*H_p_*(*y*); (**b**) *K*_1_; (**c**) *K*_2_.

Figure 5 shows the variation in the characteristics of specimens with different SCFs when the fatigue load was 20 kN and the fatigue cycle was 11,000. In this figure, *d* is the distance between the scan line and the center line.

Figure 5a shows the relationship between Δ*H_p_*(*y*) and the SCF. It could be seen that Δ*H_p_*(*y*) increased as the SCF increased from 2 to 4, but Δ*H_p_*(*y*) increased much more when the SCF increased from 3 to 4 than when the SCF increased from 2 to 3. When the SCF increased from 4 to 5, Δ*H_p_*(*y*) varied differently: the Δ*H_p_*(*y*) of both *d* = 0 and 12 mm decreased, but that of *d* = 12 mm was more obvious, and the Δ*H_p_*(*y*) of *d* = 5, 10, and 11 mm increased slightly.

The relationship between *K*_1_ and the SCF was different from that between Δ*H_p_*(*y*) and the SCF, as shown in Figure 5b. All *K*_1_ values increased as the SCF increased, but they decreased slightly when the SCF increased from 4 to 5 at *d* = 0 mm. In addition, the increases were different, and that of the SCF from 3 to 4 was the highest.

The relationship between *K*_2_ and the SCF also varied differently, as shown in Figure 5c. All the *K*_2_ values of *d* = 10, 11, and 12 mm increased as the SCF increased, but the *K*_2_ of *d* = 0 and 5 mm increased slightly at first and then remained constant.

Furthermore, it is evident from Figure 5 that all characteristics were less noticeable. The Δ*H_p_*(*y*), *K*_1_, and *K*_2_ of *d* = 0 mm and *d* = 5 mm were much smaller than those of d = 10, 11, and 12 mm. The reason for this can be found in Figure 1: the lines of *d* = 0 mm and *d* = 5 mm lay beyond the pre-cut notch, the lines of *d* = 10 mm were right across the notch root, and the lines of *d* = 11 and 12 mm just crossed the notch.

According to Figure 5, it can be found that no obvious mathematical model could be established between the SCF and any of the characteristics. Therefore, other methods should be explored, and the artificial neural network is one of the most promising methods which attracted the authors’ interests.

## 4. SCF Recognition by BP Neural Network

### 4.1. Data Sets for BP Neural Network Development and Pre-Processing

According to the results proposed above, four variables were selected as the input vector, including *L* (the maximum load), Δ*H_p_*(*y*) (the peak-to-peak value of abnormal peaks), *K*_1_ (the slope coefficient of the fitting line of *H_p_*(*y*)), and *K*_2_ (the slope coefficient of the fitting line of the curve between the abnormal peaks), and the SCF of the specimen was the only output of the BP neural network established in this study.

Numerically, the variables ranged from 10 to 40 for L, 9.17 to 25.52 for Δ*H_p_*(*y*), 1.99 to 14.33 for *K*_1_, and 4.99 to 61.42 for *K*_2_. The output values of the SCF ranged from 2 to 5. In order to accelerate the convergence and learning rate of BP neural network training and to reduce the chance of choking the BP neural network, the input variables were first normalized. The typical range is −1 to 1 or 0 to 1. In this study, the data sets were scaled to a range of 0 to 1 before submission to the BP neural network, and the formula used was as follows:

(1) $$x_{i}^{\prime }=(x_{i}-Min_{i})/(Max_{i}-Min_{i})$$

where $Max_{i}$ and $Min_{i}$ are the maximum and the minimum values within the independent variable.

All 60 data points were grouped into training and testing sets; half of them (30 data points) were used for training, and the rest (30 data points) were used to test the developed BP neural network.

### 4.2. BP Neural Network Architecture

The transfer function employed in the hidden layer was a binary sigmoid function, which is widely used for the continuous activation function with a range of 0 to 1. The function is as follows:

(2) $$F(x)=1/(1+e^{-x})$$

Since the output was already normalized to 0 to 1, the transfer function of the output layer was a log-sigmoid function:

(3) $$F(x)=(1-e^{-x})/(1+e^{-x})$$

Generally, a BP neural network comprises three layers: an input layer, hidden layer, and output layer. As mentioned above, the input layer has four input nodes such as *L*, Δ*H_p_*(*y*), *K*_1_, and *K*_2_, and the output layer only has one node, the SCF of the specimens. The selection of node number in the hidden layer is critical to the performance of the BP neural network. The basic principle is to select the number of hidden nodes that results in the smallest sum of squared errors (SSE). An empirical formula was given by Kolmogorov:

(4) $$N=\sqrt{m+n}+a$$

where *m* and *n* are the number of input and output nodes, and *a* is a constant that ranges from 1 to 10.

Eight nodes in the hidden layer were selected according to Table 3 with the smallest SSE of 0.00465 and 181 epochs when the training algorithm was traingdx.

For all numerical examples studied, the general structure of a BP neural network model with four inputs, one output, and a hidden layer of eight neurons was created.

**Figure 6:**
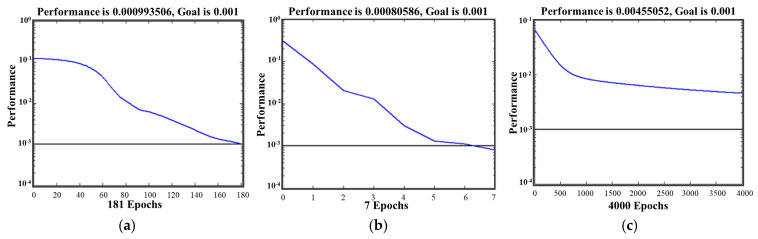
Training results of different training algorithms. (**a**) traingdx; (**b**) trainlm; (**c**) traingd.

The learning rate and SSE target were 0.1 and 0.01, respectively, taking into account the accuracy and operating time of the BP neural network. And the Levenberg–Marquat back-propagation algorithm (trainlm) was used in accordance with Figure 6 compared to other commonly used training algorithms.

### 4.3. SCF Recognition Results

**Figure 7:**
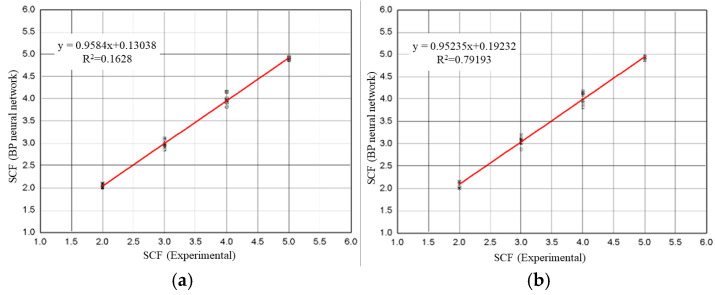
SCF values detected by BP neural network compared to experimental values: (**a**) training sets; (**b**) test sets.

Figure 7a shows the comparison of the SCF from experimental data with the SCF, which was proven using the established BP neural network. The red line represents a perfect correlation between experimental fatigue life and the fatigue life recognized by the BP neural network. Figure 7b shows a comparison between the SCF from experimental data and the SCF detected using the BP neural network.

Figure 7 shows a perfect correlation between the experimental SCF and the SCF proven by the BP neural network, meaning that it is feasible to determine the stress concentration degree of specimens based on the BP neural network established in this research.

## 5. Conclusions

The *H_p_*(*y*) of specimens with different SCFs during the tension–tension fatigue test was collected, and three characteristics, Δ*H_p_*(*y*), *K*_1_, and, *K*_2_, were extracted and analyzed during the test. A BP neural network was developed to differentiate the SCF of the specimens. The results are as follows.

(1) Δ*H_p_*(*y*), *K*_1_, and *K*_2_ were extracted as the characteristics of the MMMT signals. They changed only slightly as the fatigue cycle number increased, but they increased significantly as fatigue load increased, meaning that the MMMT signal could be affected by fatigue load much more easily than the fatigue cycle number.
(2) Δ*H_p_*(*y*), *K*_1_, and *K*_2_ all increased as the distance between the scan line and the center line increased, and the maximum load during fatigue can easily be distinguished by *K*_1_, but none of them could clearly determine the fatigue cycle number.
(3) It is not easy to differentiate SCFs based only on the characteristics mentioned in this article, but a well-established BP neural network could accurately detect the SCFs of specimens.

## Figures and Tables

**Table 1:** Chemical composition (wt.%) of 45CrNiMoVA steel.

C	Si	Mn	P	S	Cr	Ni	Mo	V
0.42~0.49	0.17~0.37	0.50~0.80	≤0.030	≤0.030	0.80~1.10	1.30~1.80	0.20~0.30	0.10~0.20

**Table 2:** Parameters of pre-cut notches of specimens (in mm) with different SCFs.

SCF	2	3	4	5
R	5.3	1.5	0.75	0.45
2a	10.6	3	1.5	0.9
b	5	4.5	5	5.25

**Table 3:** Network performance and epochs with different hidden nodes.

Nodes	4	5	6	7	8	9	10	11	12	13
SSE	0.00591	0.00534	0.00623	0.00564	0.00465	0.00479	0.00527	0.00555	0.00498	0.00602
Epochs	974	596	632	478	181	186	265	285	380	256

## Data Availability

The original contributions presented in this study are included in the article. Further inquiries can be directed to the corresponding author.
